# Midterm Outcome After Septal Myectomy and Medical Therapy in Mildly Symptomatic Patients With Hypertrophic Obstructive Cardiomyopathy

**DOI:** 10.3389/fcvm.2022.855491

**Published:** 2022-03-25

**Authors:** Jiejun Sun, Lin Liang, Peijin Li, Tengyong Jiang, Xianpeng Yu, Changwei Ren, Ran Dong, Jiqiang He

**Affiliations:** ^1^Department of Cardiology, Beijing Anzhen Hospital, Beijing Institute of Heart, Lung and Blood Vessel Diseases, Capital Medical University, Beijing, China; ^2^Department of Cardiovascular Surgery Center, Beijing Anzhen Hospital, Beijing Institute of Heart, Lung and Blood Vessel Diseases, Capital Medical University, Beijing, China

**Keywords:** hypertrophic obstructive cardiomyopathy (HOCM), mild symptom, septal myectomy, medical therapy (MT), outcome

## Abstract

**Objective:**

The purpose of this study was mainly to determine the midterm outcome of septal myectomy (SM) and medical therapy (MT) in mildly symptomatic patients (NYHA class II) with hypertrophic obstructive cardiomyopathy (HOCM).

**Methods:**

The study cohort consisted of 184 mildly symptomatic patients with HOCM evaluated in Beijing Anzhen Hospital, Capital Medical University between March 2001 and December 2017, including 82 patients in the SM group and 102 patients in the MT group. Overall survival and HCM-related survival were mainly observed.

**Results:**

The average follow-up time was 5.0 years. Compared to patients accepting MT, patients treated with SM were associated with comparable overall survival (96.5% and 93.1% vs. 92.9% and 83.0% at 5 and 10 years, respectively; *P* = 0.197) and HCM-related survival (98.7% and 98.7% vs. 94.2% and 86.1% at 5 and 10 years, respectively; *P* = 0.063). However, compared to MT, SM was superior at improvement of NYHA class (1.3 ± 0.6 vs. 2.1 ± 0.5, *P* < 0.001) and mean reduction of resting left ventricular outflow (LVOT) gradient (78.5 ± 18.6% vs. 28.3 ± 18.4%, *P* < 0.001). Multivariate analysis suggested that resting LVOT gradient in the last clinical examination was an independent predictor of all-cause mortality (HR = 1.017, 95%CI: 1.000–1.034, *P* = 0.045) and HCM-related mortality (HR = 1.024, 95%CI: 1.005–1.043, *P* = 0.012) in the entire cohort.

**Conclusion:**

Compared with MT, SM had comparable overall survival and HCM-related survival in mildly symptomatic HOCM patients, but SM had advantages on improving clinical symptoms and reducing resting LVOT gradient. Resting LVOT gradient in the last clinical examination was an independent predictor of all-cause mortality and HCM-related mortality.

## Introduction

Hypertrophic obstructive cardiomyopathy (HOCM) is a genetic heart disease characterized by marked cardiomyocyte hypertrophy and left ventricular outflow tract (LVOT) obstruction (1–3). LVOT obstruction not only leads to exertional dyspnea, fatigue, chest pain, and limited exercise capacity (4), but also increases all-cause mortality and the incidence of sudden cardiac death (SCD) in patients with hypertrophic cardiomyopathy (HCM) (5, 6). Septal myectomy (SM) has been proven by multiple studies that provides excellent long-term survival and freedom from recurrent symptoms in highly symptomatic patients with HOCM (7, 8). Currently, SM is mainly recommended for HOCM patients with severe symptoms (NYHA class III-IV or recurrent exertional syncope) despite optimal medical therapy (MT) in European and American clinical practice guidelines (Class I indication) (9, 10). However, one study indicated that in mildly symptomatic (NYHA class II) or asymptomatic patients with HOCM, severity of LVOT gradient at rest was independently associated with a higher risk of developing heart failure and death (11). In our recent research, we have demonstrated that although alcohol septal ablation (ASA) did not provide better long-term survival in mildly symptomatic HOCM patients compared with MT, ASA could evidently improve clinical symptoms and reduce LVOT gradient, which may be a kind of reasonable alternative for patients intolerant to MT (12). Nevertheless, there is no definite evidence for SM applying to mildly symptomatic HCM patients with severe obstruction. Recently, a study made by Alashi et al. (13) suggested that in patients with HOCM, earlier surgery vs. surgery for guideline-based Class I indication had a higher long-term survival, which was similar to the age- and sex-matched US population. However, there is no study that directly compares the outcome of SM and MT in mildly symptomatic patients with HOCM. Therefore, this study was conducted to primarily evaluate the outcome of SM in mildly symptomatic patients with HOCM, as a comparison with MT.

## Materials and Methods

### Study Patients

This retrospective study consisted of 184 mildly symptomatic patients with HOCM from Beijing Anzhen Hospital, Capital Medical University between March 2001 and December 2017, including 102 patients in the MT group and 82 patients in the SM group. Informed consent was obtained from each patient before the study began. This study was conducted according to the ethical standards of Helsinki Declaration, Chinese clinical practice regulations and guidelines, and rules of Medicine Ethics Committee of Beijing Anzhen Hospital (institutional review board number: No. 2020087x, date of approval: December 22, 2020). This study has been registered on the Chinese Clinical Trial Registry (No. ChiCTR2000041464). Each patient was adult (age ≥ 18 years) and had an established diagnosis of HOCM. In an adult, the diagnosis of HOCM was made as described formerly as follows (9, 10, 14): (1) the wall thickness of one or more left ventricular myocardial segments measured by any imaging technique was ≥15 mm and without any other disease accounting for cardiomyocyte hypertrophy; (2) LVOT gradient ≥ 30 mmHg at rest or after provocation caused by anterior systolic displacement of mitral valve. The MT group consisted of mildly symptomatic HOCM patients who have obtained optimal MT (maximum tolerable dose of beta-receptor antagonists and/or calcium channel blockers). Consecutive patients met the following criteria were enrolled in the SM group, including: (1) intolerant to medical treatments (After taking β-receptor antagonist or calcium channel blocker, the patient had obvious symptoms such as hypotension-related dizziness or evidently fatigue, which can significantly reduce the patients’ quality of life); (2) had a strong wish for symptomatic relief; (3) LVOT gradient ≥ 50 mmHg at rest or after provocation. Patients met the following criteria were excluded from the SM groups: (1) patients with severe comorbidities, such as severe hepatic and/or renal dysfunction, malignant tumors; (2) patients with complete right bundle branch block; (3) patients with high risk of SCD, for example: recorded exertional syncope, family history of premature SCD and non-sustained ventricular tachycardia; (4) patients have been treated with ASA. All patients were informed about potential risk of SM and agreed with the procedure. Procedure of SM have been described in detail on several previous reports (15–19).

### Follow-Up

In the MT group, follow-up started at the first clinic contact of patients after March 1, 2001 in Beijing Anzhen Hospital; in the SM group, follow-up started on the day of surgical intervention. If no endpoints occurred during follow-up, follow-up ended at the last check-up and the final censoring date was set at April 1, 2021. Follow-up was conducted by means of clinic visit, telephone contact and online communication. The following parameters were documented: symptoms, arrhythmic events and pacemaker implantation, causes of death (confirmed by reviewing the medical records and national registries of deaths or communicating with family members of the patients), electrocardiography and echocardiographic parameters.

### Endpoints

The primary and secondary endpoints of this study were all-cause mortality and HCM-related death, respectively. In addition, we want to determined: (1) predictors of all-cause mortality and HCM-related mortality; (2) difference of symptom improvement, occurrence of new-onset atrial fibrillation (AF) and echocardiographic parameters at the last check-up between two groups. HCM-related death was defined as death caused by either SCD, congestive heart failure (CHF) or AF-related stroke (20). SCD was defined as instant and death unexpected within 1 h after a witnessing collapse in patients who previously were in a stable clinical condition, or nocturnal death with no antecedent history of worsening symptoms (21). Death caused by CHF was defined as death that occurred in context of progressive cardiac decompensation due to development of pulmonary edema or cardiogenic shock (21).

### Statistical Analysis

All statistical analyses were done with SPSS 25.0 (IBM, Armonk, NY, United Status) and GraphPad Prism 8.0 (GraphPad Software Inc., La Jolla, CA, United States). Normally distributed measurement data are expressed as mean ± SD and non-normally distributed continuous data as median [interquartile range (IQR)]. In order to compare continuous variables, two independent sample *t*-test was used between two groups, while paired *t*-test was used within the same group. The chi-square or Fisher’s exact test was used to compare non-continuous variables expressed as numerals (percentages). The Kaplan–Meier method with log-rank test was used to determine and compare the cumulative survival of different groups. To identify the prognostic predictors of all-cause mortality and HCM-related mortality, Cox regression model was used. First, the potential variables may affect all-cause mortality and HCM-related mortality were evaluated in a univariable model. Second, input variables with *P* < 0.10 into the backward stepwise multivariate analysis. It was considered statistically significant if *P*-values (2-sided) were less than 0.05.

## Results

### Baseline Characteristics

Table 1 lists the baseline characteristics of 184 patients. Of these 184 patients, 102 were treated by medication (e.g., beta-receptor antagonists, calcium channel blockers) and 82 underwent SM. Patients in the SM group were younger (48.9 ± 10.4 years) than those in the MT group (55.1 ± 14.8 years, *p* = 0.001). Compared with MT group, the rate of comorbidity (particularly coronary artery disease and hypertension) of SM group was lower (26.8% vs. 52.9%, *p* < 0.001). LA diameter of the SM group was larger than that of the MT group (42.7 ± 7.3 vs. 39.5 ± 6.1 mm, *p* = 0.001). LVOT gradient at rest of SM group was 89.1 ± 35.7 mmHg, and it was obviously larger than that of MT group (66.3 ± 35.0 mmHg, *p* < 0.001).

**TABLE 1:** Characteristics of 184 mildly symptomatic patients with HOCM at baseline.

Variable	MT (*n* = 102)	SM (*n* = 82)	*P*-value
Age (yrs)	55.1 ± 14.8	48.9 ± 10.4	0.001
Female (n,%)	43.0 (42.2)	32.0 (38.6)	0.667
BMI (kg/m^2^)	25.8 ± 3.8	24.9 ± 3.4	0.128
SBP (mmHg)	123.7 ± 16.0	123.0 ± 15.8	0.797
DBP (mmHg)	74.4 ± 10.5	72.9 ± 10.7	0.339
Comorbidity (n,%)	54.0 (52.9)	22.0 (26.8)	<0.001
Coronary artery disease	18.0 (17.6)	5.0 (6.1)	0.019
Hypertension	41.0 (40.2)	17.0 (20.7)	0.005
Diabetes	7.0 (6.9)	2.0 (2.4)	0.299
HCM family history (n,%)	7.0 (6.9)	5.0 (6.1)	0.834
History of HOCM (yrs)	3.3 ± 4.5	3.7 ± 3.8	0.593
History of AF (n,%)	6.0 (5.9)	8.0 (9.8)	0.325
NYHA class	2.0 ± 0.0	2.0 ± 0.0	–
LA diameter (mm)	39.5 ± 6.1	42.7 ± 7.3	0.001
LV end-diastolic diameter (mm)	43.0 ± 5.0	43.6 ± 5.1	0.388
LV ejection fraction (%)	69.4 ± 7.5	69.0 ± 6.8	0.714
Septal thickness (mm)	20.5 ± 5.0	21.0 ± 5.3	0.593
Resting LVOT gradient (mmHg)	66.3 ± 35.0	89.1 ± 35.7	<0.001

*MT, medical therapy; SM, septal myectomy; BMI, body mass index; SBP, systolic blood pressure; DBP, diastolic blood pressure; HCM, hypertrophic cardiomyopathy; HOCM, hypertrophic obstructive cardiomyopathy; AF, atrial fibrillation; NYHA, New York Heart Association; LA, left atrium; LV, left ventricular; LVOT, left ventricular outflow tract.*

### Procedure Data

A total of 82 patients underwent SM. There were five patients (5/82, 6.1%) died during the perioperative period in the SM group: four of them died of CHF, and one died of cerebral hemorrhage. In the perioperative period, one patient (1/82, 1.2%) implanted a permanent pacemaker due to third-degree atrioventricular block after SM.

### Survival

**FIGURE 1:**
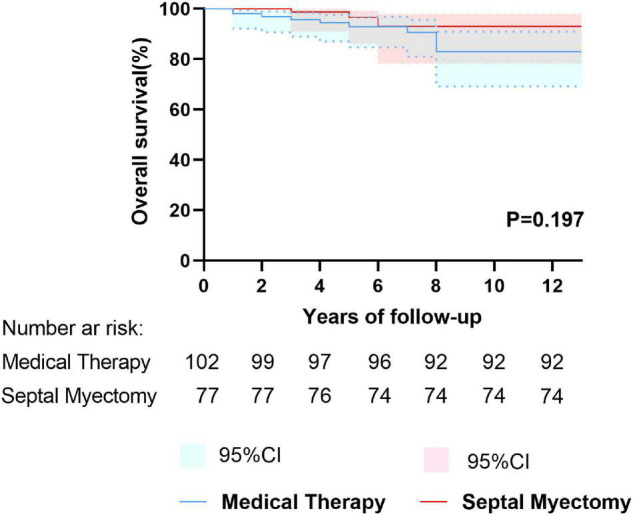
Kaplan–Meier curves depicting overall survival between the septal myectomy and medical therapy groups.

**FIGURE 2:**
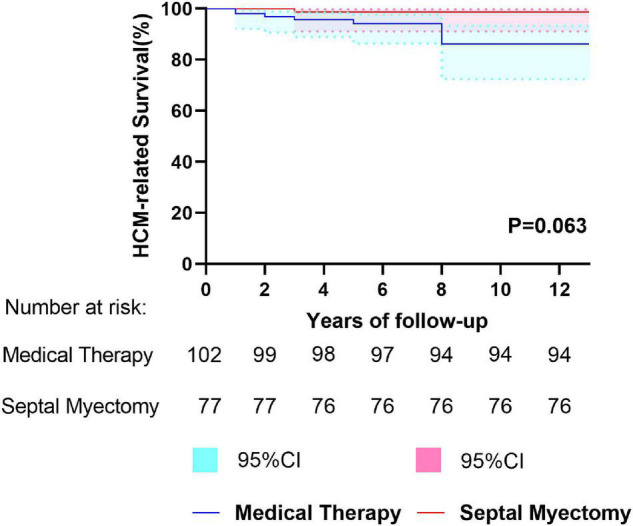
Kaplan–Meier curves depicting HCM-related survival between the septal myectomy and medical therapy groups.

Follow-up was completed in 179 patients and the median follow-up time was 5.0 years (IQR: 4.0 to 8.0 years, maximum: 18.0 years). In the period of follow-up, no patients accepted septal reduction therapy or implanted cardioverter defibrillator, either in the MT or SM groups. There were 14 deaths in the entire cohort during follow-up, including 11 deaths in the MT group (annual mortality rate: 0.6%/year) and 3 deaths in the SM group (annual mortality rate: 0.3%/year). The clinical endpoints of patients are summarized in Table 2. Cardiovascular death accounted for larger percentage (10/14, 71.4%) of all-cause death in this study, including 9 (9/11, 81.8%) in the MT group and 1 (1/3, 33.3%) in the SM group. There were 9 (9/102, 8.8%) patients died of HCM-related death in the MT group: 5 (5/102, 4.9%) due to SCD, 1 (1/102, 1.0%) due to CHF and 3 (3/102, 2.9%) due to AF-related stroke. However, in the SM group, only 1 (1/77, 1.3%) died of HCM-related death during the long-term follow-up, and the remaining 2 patients died of severe pneumonia (5 years after SM) and malignant tumors (6 years after SM), respectively. 5-year and 10-year overall survival of the SM group was 96.5% (95%CI: 91.6% to 100.0%) and 93.1% (95%CI: 84.9% to 100.0%), respectively. This survival was comparable to that of the MT group, whose 5- and 10-year overall survival were 92.9% (95%CI: 87.4% to 98.4%) and 83.0% (95%CI: 72.4% to 93.6%), respectively (*P* = 0.197) (Figure 1). 5- and 10-year HCM-related survival for two groups was 98.7% (95%CI: 96.2% to 100.0%) and 98.7% (95%CI: 96.2% to 100.0%) vs. 94.2% (95%CI: 89.1% to 99.3%) and 86.1% (95%CI: 76.3% to 95.9%), respectively (*P* = 0.063) (Figure 2). Cox multivariate regression analysis suggested that resting LVOT gradient in the last clinical examination was an independent predictor of all-cause mortality (HR = 1.017, 95%CI: 1.000–1.034, *P* = 0.045) and HCM-related mortality (HR = 1.024, 95%CI: 1.005–1.043, *P* = 0.012) (Table 3 and Table 4).

**TABLE 2:** The classification of clinical endpoints during follow-up (n,%).

Variable	MT (*n* = 102)	SM (*n* = 77)	*P*-value
All-cause death	11 (10.8)	3 (3.9)	0.089
Cardiovascular death	9 (8.8)	1 (1.3)	0.066
Non-cardiovascular death	2 (2.0)	2 (2.6)	1.000
5-year overall survival	92.9%	96.5%	–
10-year overall survival	83.0%	93.1%	–
HCM-related death	9 (8.8)	1 (1.3)	0.066
SCD	5 (4.9)	0	–
CHF	1 (1.0)	1 (1.3)	1.000
AF-related stroke	3 (2.9)	0	–
5-year HCM-related survival	94.2%	98.7%	–
10-year HCM-related survival	86.1%	98.7%	–

*MT, medical therapy; SM, septal myectomy; HCM, hypertrophic cardiomyopathy; SCD, sudden cardiac death; CHF, congestive heart failure; AF, atrial fibrillation.*

**TABLE 3:** Predictors of all-cause mortality.

Variable	Univariate		Multivariate	
	HR (95%CI)	*P*-value	HR (95%CI)	*P*-value
**Baseline**				
Age (yrs)	1.035 (0.991–1.082)	0.120	–	–
Female	1.380 (0.463–4.112)	0.563	–	–
History of AF (n,%)	2.247 (0.496–10.175)	0.293		
SM	0.438 (0.120–1.602)	0.212	–	–
LA diameter (mm)	1.002 (0.926–1.085)	0.952	–	–
LV end-diastolic diameter (mm)	0.952 (0.858–1.056)	0.350	–	–
LV ejection fraction (%)	0.980 (0.909–1.058)	0.609	–	–
Septal thickness (mm)	1.006 (0.893–1.132)	0.924	–	–
Resting LVOT gradient (mmHg)	0.999 (0.984–1.132)	0.929	–	–
**Follow up**				
New-onset AF	0.729 (0.161–3.293)	0.670	–	–
LA diameter (mm)	1.040 (0.967–1.120)	0.291	–	–
LV end-diastolic diameter (mm)	0.911 (0.816–1.017)	0.097	0.942 (0.836–1.062)	0.331
LV ejection fraction (%)	1.081 (1.000–1.168)	0.049	1.050 (0.969–1.138)	0.230
Septal thickness (mm)	1.052 (0.932–1.186)	0.414	–	–
Resting LVOT gradient (mmHg)	1.020 (1.003–1.036)	0.022	1.017 (1.000–1.034)	0.045

*HR, hazard ratio; CI, confidence interval; AF, atrial fibrillation; SM, septal myectomy; LA, left atrium; LV, left ventricular; LVOT, left ventricular outflow tract.*

**TABLE 4:** Predictors of HCM-related mortality.

Variable	Univariate		Multivariate	
	HR (95%CI)	*P*-value	HR (95%CI)	*P*-value
**Baseline**				
Age (yrs)	1.041 (0.998–1.098)	0.132	–	–
Female	2.064 (0.554–7.700)	0.280	–	–
History of AF (n,%)	3.720 (0.770–17.975)	0.102		
SM	0.175 (0.022–1.411)	0.102		–
LA diameter (mm)	0.982 (0.893–1.080)	0.710	–	–
LV end-diastolic diameter (mm)	0.965 (0.851–1.093)	0.575	–	–
LV ejection fraction (%)	0.969 (0.885–1.060)	0.488	–	–
Septal thickness (mm)	1.006 (0.878–1.153)	0.931	–	–
Resting LVOT gradient (mmHg)	1.001 (0.983–1.019)	0.952	–	–
**Follow up**				
New-onset AF	1.136 (0.235–5.480)	0.874	–	–
LA diameter (mm)	1.043 (0.956–1.138)	0.347	–	–
LV end-diastolic diameter (mm)	0.943 (0.828–1.073)	0.372	–	
LV ejection fraction (%)	1.097 (0.996–1.209)	0.059	1.079 (0.982–1.185)	0.113
Septal thickness (mm)	1.086 (0.969–1.217)	0.156	–	–
Resting LVOT gradient (mmHg)	1.027 (1.007–1.046)	0.007	1.024 (1.005–1.043)	0.012

*HR, hazard ratio; CI, confidence interval; AF, atrial fibrillation; SM, septal myectomy; LA, left atrium; LV, left ventricular; LVOT, left ventricular outflow tract.*

### Clinical Outcome

Table 5 lists the clinical results of 179 patients. In the SM group, the clinical symptoms were remarkably improved (NYHA class post-SM: 1.3 ± 0.6, *P* < 0.001), and 56 patients (56/77, 72.7%) were in NYHA class I. Nevertheless, the patients’ clinical symptoms did not improve after MT (NYHA class after medical treatment: 2.1 ± 0.5, *P* = 0.127). Resting LVOT gradient, with an average decrease of 78.5%, had reduced from 89.4 ± 35.5 to 16.7 ± 12.2 mmHg (*p* < 0.001) after SM. Moreover, there were 67 patients (67/77, 87.0%) after SM with a resting LVOT gradient < 30 mmHg. Meanwhile, the resting LVOT gradient of MT group, with an average decrease of 28.3%, was reduced from 66.3 ± 35.0 to 56.5 ± 27.7 mmHg (*P* = 0.001). However, there were only 22 patients (22/102, 21.6%) in the MT group with a resting LVOT gradient < 30 mmHg. Patients of the SM group had LA diameter reducing from 42.7 ± 7.3 to 37.7 ± 5.2 mm (*P* < 0.001). Instead, patients of MT group had left atrium diameter increasing from 39.5 ± 6.1 to 43.2 ± 6.9 mm (*P* < 0.001). During the period of following up, two patients (2/77, 2.6%) implanted permanent pacemaker due to third-degree atrioventricular block after SM.

**TABLE 5:** Clinical and echocardiographic characteristics of 179 mildly symptomatic patients with HOCM at the last check-up.

Variable	MT (*n* = 102)	SM (*n* = 77)	*P*-value
NYHA class	2.1 ± 0.5	1.3 ± 0.6^b^	<0.001
NYHA class I (n,%)	7.0 (6.0)	56.0 (72.7)	<0.001
NYHA class II (n,%)	81.0 (79.4)	17.0 (22.1)	<0.001
NYHA class III (n,%)	14.0 (13.7)	3.0 (3.9)	0.026
NYHA class IV (n,%)	0.0 (0.0)	1.0 (1.3)	0.430
NYHA class III/IV (n,%)	14.0 (13.7)	4.0 (5.2)	0.060
New-onset AF (n,%)	20 (20.8)	13 (18.8)	0.752
LA diameter (mm)	43.2 ± 6.9^b^	37.7 ± 5.2^b^	<0.001
LVend-diastolic diameter (mm)	43.8 ± 4.9	42.9 ± 5.4	0.238
LV ejection fraction (%)	66.2 ± 7.2^b^	62.7 ± 6.8^b^	0.001
Septal thickness (mm)	19.9 ± 4.4	17.8 ± 4.6^b^	0.002
Resting LVOT gradient (mmHg)	56.5 ± 27.7^a^	16.7 ± 12.2^b^	<0.001
Reduction in LVOT gradient (%)	28.3 ± 18.4	78.5 ± 18.6	<0.001

*MT, medical therapy; SM, septal myectomy; NYHA, New York Heart Association; AF, atrial fibrillation; LA, left atrium; LV, left ventricular; LVOT, left ventricular outflow tract; ^a^P < 0.01 and ^b^P < 0.001 compared with the baseline characteristics.*

## Discussion

This study firstly and directly compares the outcome of SM and MT in mildly symptomatic patients with HOCM. The crucial findings of this study were listed as follows: (1) overall survival and HCM-related survival of SM group were comparable to those of MT group; (2) compared to the MT, SM had advantages on improving clinical symptoms and reducing resting LVOT gradient; (3) resting LVOT gradient at the last clinical check-up was an independent predictor of all-cause mortality and HCM-related mortality in mildly symptomatic patients with HOCM.

Now, SM has been proven by multiple previous studies that exerts a positive effect on long-term prognosis for HOCM patients with severe symptoms (7, 8, 22). It is worth noting that a recent study conducted by Desai and his colleagues concerning a large proportion of mildly symptomatic or asymptomatic patients (88% patients with NYHA class I/II) with HOCM demonstrated that the composite event (death except non-cardiac causes and/or appropriate ICD discharge) rate of MT group was twice as high as that of SM group (76% patients with NYHA class I/II) (23). However, compared with MT, whether early surgery could provide better survival for mildly symptomatic patients with HOCM is not yet known. Therefore, the present study was dedicated to discuss this issue.

In our study, 10-year overall survival of patients in MT group was 83.0%. Similar to our data, a study conducted by Vriesendorp et al. (24) reported a 10-year overall survival of 84.0% of MT group, but their study cohort were mildly symptomatic or asymptomatic (NYHA class I/II) patients with HOCM. Our survival rate of MT group was higher compared with two other studies with 10-year overall survival of 75.8% (Ball et al., 33.3% patients of MT group in NYHA class III/IV) (25) and 72.2% (Yin-Jian Yang et al., 44.4% patients of MT group in NYHA class III/IV) (26). This might be due to the fact that these two studies involved some HOCM patients with NYHA class III/IV in the MT group, and multiple studies have demonstrated that for patients with HOCM, NYHA class III/IV is independently associated with worse prognosis (8, 27). In the present study, mildly symptomatic patients with HOCM after SM had comparable overall survival and HCM-related survival to those treated with medication. But compared with MT, SM had obvious advantages on maintaining long-lasting improvement in symptoms. Our data indicated that the clinical symptoms was remarkably improved after SM (NYHA class post-SM: 1.3 ± 0.6, *P* < 0.001), and 56 patients (72.7%) were in NYHA class I. However, the patients’ clinical symptoms did not improve after MT (NYHA class after medical treatment: 2.1 ± 0.5, *P* = 0.127). Furthermore, our data indicated that 10-year overall survival and HCM-related survival of SM group were 93.1% and 98.7%, respectively, which evidently higher than those reported by Ball et al. (25) concerning HOCM patients some with severe symptoms (33.3% patients with NYHA class III/IV) in the MT group (10-year overall survival and HCM-related survival 75.8 and 86.9%, respectively). Recently, outcome of earlier surgery vs. surgery for guideline-based Class I indication in patients with HOCM was discussed by Alashi et al. (13). In their study, earlier surgery was applied to patients who were in NYHA class II with drug intolerance or who were in NYHA class I but with symptomatic impairment of exercise capacity despite optimal medical therapy. The data of Alashi et al. (13) indicated that for patients with HOCM, earlier surgery vs. surgery for Class I indication was associated with a higher long-term survival, close to the age- and sex- matched US population. Therefore, combined the above important findings, we considered that earlier surgical intervention may be a reasonable option for mildly symptomatic patients with HOCM who intolerant to MT, rather than only undergoing watchful waiting.

Multiple studies have demonstrated that the prognosis of HCM patients with obstruction is poorer than that of those without obstruction, especially for patients with severe symptoms (7, 28, 29). Moreover, a study conducted by Sorajja et al. (11) suggested that for mildly symptomatic or asymptomatic HOCM patients, an elevated LVOT gradient was independently associated with higher risk of developing heart failure and death. Similarly, results of our study suggested that resting LVOT gradient in the last clinical examination was an independent predictor of all-cause mortality in mildly symptomatic patients with HOCM, and every 1 mmHg increase added the risk of all-cause mortality by 1.7%. Additionally, our data suggested that resting LVOT gradient in the last clinical examination was also an independent predictor of HCM-related mortality, and every 1 mmHg increase added the risk of HCM-related mortality by 2.4%. Additionally, in multiple clinical trials, SM has been proven to be able to safely and effectively reduce the LVOT gradient in patients with HCM (8, 30, 31). Likewise, our research results also confirmed this point. Moreover, the present study indicated that compared with MT, SM had advantages on reducing resting LVOT gradient (average decrease on resting LVOT gradient: 78.5% vs. 28.3%, *P* < 0.001). Consequently, in order to reduce the negative impact of high LVOT gradient, SM is also seemed to be a reasonable choice for mildly symptomatic patients with high LVOT gradient who intolerant to drug treatments.

There are several limitations in this study. First, this was a retrospective study with a small sample from a single-center, a relatively experienced HCM management center in China. Therefore, our results were limited by referral and selection bias and might not be generalizable to else centers. Second, in this study, level of symptoms of patients was based on self-statement. However, some patients may adapt themselves to their restriction of exercise capacity and thus report a lower degree of symptom severity, which may influence the baseline characteristics and clinical results of follow-up in this study. Third, an advantage of this research was that we compared the prognosis of conservative treatment and surgical intervention, but overall survival and HCM-related survival of patients enrolled in this research did not make a comparison with expected survival of an age- and sex-matched Chinese general population.

## Conclusion

Compared with MT, SM had comparable overall survival and HCM-related survival in mildly symptomatic HOCM patients, but SM had advantages on improving clinical symptoms and reducing resting LVOT gradient. Resting LVOT gradient in the last clinical examination was an independent predictor of all-cause mortality and HCM-related mortality.

## Data Availability Statement

The data that support the findings of this study are available on request from the corresponding author. The data are not publicly available due to privacy or ethical restrictions.

## Ethics Statement

The studies involving human participants were reviewed and approved by the Medicine Ethics Committee of Beijing Anzhen Hospital. Written informed consent for participation was not required for this study in accordance with the national legislation and the institutional requirements.

## Author Contributions

All authors listed have made a substantial, direct, and intellectual contribution to the work, and approved it for publication.

## Conflict of Interest

The authors declare that the research was conducted in the absence of any commercial or financial relationships that could be construed as a potential conflict of interest.

## Publisher’s Note

All claims expressed in this article are solely those of the authors and do not necessarily represent those of their affiliated organizations, or those of the publisher, the editors and the reviewers. Any product that may be evaluated in this article, or claim that may be made by its manufacturer, is not guaranteed or endorsed by the publisher.
